# “Protocol for a phase 2, randomized, double-blind, placebo-controlled, safety and efficacy study of dutogliptin in combination with filgrastim in early recovery post-myocardial infarction”: study protocol for a randomized controlled trial

**DOI:** 10.1186/s13063-020-04652-0

**Published:** 2020-08-26

**Authors:** Dirk von Lewinski, Joseph B. Selvanayagam, Richard A. Schatz, Bernd Jilma, Jacek Kubica, Thomas J. Povsic, Darrell Nix, Stephan Henauer, Markus Wallner, on behalf of the REC-DUT-002 study group

**Affiliations:** 1 Department of Cardiology, Medical University of Graz, Graz, Austria; 2 Department of Cardiovascular Medicine, Flinders University of South Australia, South Australian Health and Medical Research Institute, Adelaide, Australia; 3 Gene and Cell Therapy, Scripps Clinic, La Jolla, CA USA; 4 Department of Clinical Pharmacology, Medical University of Vienna, Vienna, Austria; 5 Nicolaus Copernicus University, Bydgoszcz, Poland; 6 Duke Clinical Research Institute and Duke Medicine, Duke University, Durham, NC 27705 USA; 7 RECARDIO Inc., San Francisco, CA USA; 8 Cardiovascular Research Center, Lewis Katz School of Medicine, Temple University, Philadelphia, PA USA; 9 Center for Biomarker Research in Medicine, CBmed GmbH, Graz, Austria

**Keywords:** Filgrastim, Dutogliptin, SDF-1α, Myocardial infarction, Randomized controlled trial

## Abstract

**Background:**

Regenerative therapies offer new approaches to improve cardiac function after acute ST-elevation myocardial infarction (STEMI). Previous trials using bone marrow cells, selected stem cell populations, or cardiac stem cell progenitors require invasive procedures and had so far inconclusive results. A less invasive approach utilizes granulocyte-colony stimulating factor (G-CSF) to mobilize stem cells to circulating blood and induce neovascularization and differentiation into endothelial cells and cardiomyocytes. Stromal cell-derived factor 1 alpha (SDF-1α) is an important chemokine for initiating stem cell migration and homing to ischemic myocardium. SDF-1α concentrations can be increased by inhibition of CD26/DPP4. Dutogliptin, a novel DPP4 inhibitor, combined with stem cell mobilization using G-CSF significantly improved survival and reduced infarct size in a murine model.

**Methods:**

We test the safety and tolerability and efficacy of dutogliptin in combination with filgrastim (G-CSF) in patients with STEMI (EF < 45%) following percutaneous coronary intervention (PCI). Preliminary efficacy will be analyzed using cardiac magnetic resonance imaging (cMRI) to detect > 3.8% improvement in left ventricular ejection fraction (LV-EF) compared to placebo. One hundred forty subjects will be randomized to filgrastim plus dutogliptin or matching placebos.

**Discussion:**

The REC-DUT-002 trial is the first to evaluate dutogliptin in combination with G-CSF in patients with STEMI. Results will lay the foundation for an appropriately powered cardiovascular outcome trial to test the efficacy of this combined pharmacological strategy.

**Trial registration:**

EudraCT no.: 2018-000916-75. Registered on 7 June 2018. IND number: 123717

## Administrative information

Note: the numbers in curly brackets in this protocol refer to SPIRIT checklist item numbers. The order of the items has been modified to group similar items (see http://www.equator-network.org/reporting-guidelines/spirit-2013-statement-defining-standard-protocol-items-for-clinical-trials/).

**Table:** 

Title {1}	Protocol for a phase 2, Randomized, Double Blind, Placebo-Controlled, Safety and Efficacy Study of Dutogliptin in Combination with Filgrastim in Early Recovery Post-Myocardial Infarction
Trial registration {2a and 2b}.	IND Number: 123717 EudraCT No.: 2018-000916-75
Protocol version {3}	1.3 (10 October 2019)
Funding {4}	This research is funded by the sponsor RECARDIO, Inc., 1 Market Street San Francisco, CA 94150, USA.
Author details {5a}	**Dirk von Lewinski** ^1^*, Joseph B Selvanayagam ^**2**^_**,**_ **Richard A Schatz** ^**3**^**, Bernd Jilma** ^**4**^**, Jacek Kubica** ^**5**^**, Tom Povsic** ^**6**^**, Darrell Nix** ^**7**^**, Stephan Henauer** ^**7**^**, and Markus Wallner** ^**1,8,9**^ **on behalf of the REC-DUT-002 study group** ^1^Department of Cardiology, Medical University of Graz, Austria ^2^Department of Cardiovascular Medicine, Flinders University of South Australia, South Australian Health and Medical Research Institute, Australia ^3^Gene and Cell Therapy, Scripps Clinic, La Jolla, USA ^4^Department of Clinical Pharmacology, Medical University of Vienna, Austria ^5^Nicolaus Copernicus University, Bydgoszcz, Poland ^6^Heart Center Clinical Research, Duke University, Durham, NC, USA ^7^RECARDIO Inc., San Francisco, CA, USA ^8^Cardiovascular Research Center, Lewis Katz School of Medicine Temple University, Philadelphia, PA, USA ^9^Center for Biomarker Research in Medicine, CBmed GmbH, Graz, Austria
Name and contact information for the trial sponsor {5b}	RECARDIO, Inc., 1 Market Street San Francisco, CA 94150, USA.
Role of sponsor {5c}	D.N. and S.H. are employees of the funder and were involved in designing the study and reviewing the manuscript.

## Introduction

### Background and rationale {6a}

Regenerative therapies for the treatment of patients with cardiovascular diseases have the potential to improve cardiac function, quality of life, and survival, and offer a new approach to mitigating myocardial injury after acute ST-elevation myocardial infarction (STEMI) [1]. The trials exploring the use of bone marrow stem cells for this indication have shown largely mixed or inconclusive results to date [2–5]. The use of 2nd generation stem cells, such as selected stem cell populations [6] or cardiac stem cell progenitors [7], reported promising results but has not undergone rigorous testing. Moreover, all these approaches require invasive stem cell harvest via bone marrow biopsy or apheresis and invasive intracoronary or myocardial administration. A less invasive approach is to utilize granulocyte-colony stimulating factor (G-CSF) after STEMI to increase the release of bone marrow stem cells into the circulation. G-CSF administration significantly increases circulating CD34 (cluster of differentiation) and CD31-positive hematopoietic stem cells. These stem cells induce neovascularization, and G-CSF also facilitates differentiation into endothelial cells and cardiomyocytes [8]. Despite demonstration of decreased apoptosis and improved arteriogenesis in the infarct zone, G-CSF monotherapy has been shown to be clinically ineffective in several trials [9–11]. In contrast, the recently published outcomes of the cMR substudy of the STEMI Outcome trial [12] are promising: early administration of G-CSF improved global systolic function, adverse remodeling, scar size, and myocardial strain in patients with STEMI. Nevertheless, efficacy of G-CSF monotherapy was moderate and might possibly be explained by inadequate homing and migration of the mobilized cells to the area of injury. Stromal cell-derived factor 1 alpha (SDF-1α) is an important chemokine for initiating stem cell migration and homing to cardiac sites of ischemia with consequent neovascularization, activation of residual cardioblasts, and anti-apoptotic pleiotropic effects [13]. Therefore, the local preservation of SDF-1α represents a promising approach to treat patients with STEMI. However, safety concerns and the need of invasive transplantation protocols limit strategies to augment SDF-1α gene expression or protein delivery to the ischemic myocardium [14].

**Fig. 1:**
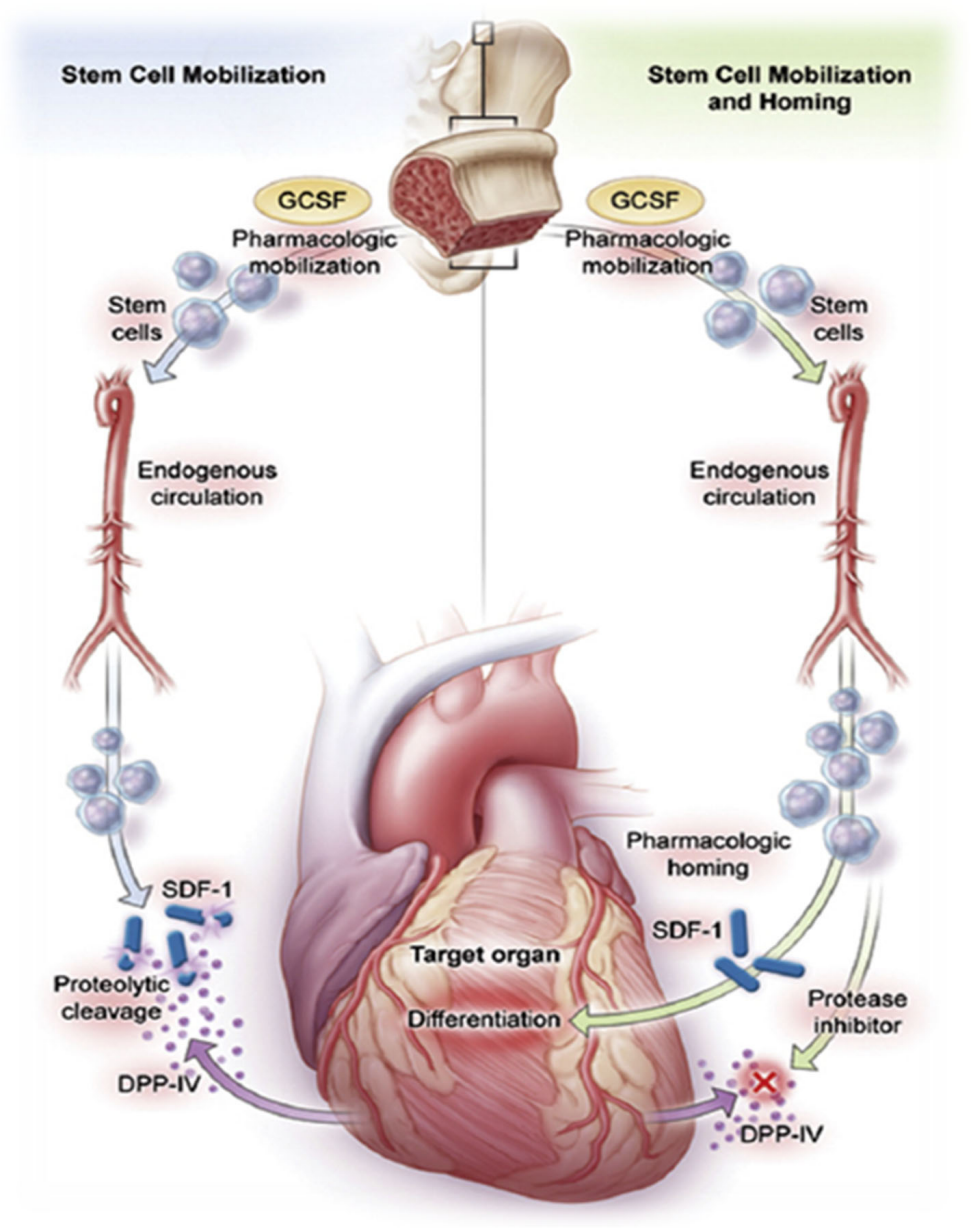
Potential beneficial mechanisms of filgrastim plus dutogliptin

An alternative approach to increase SDF-1α concentration in the injured heart is inhibition of CD26/DPP4, which is responsible for cleavage and inactivation of SDF-1α. Early preclinical data demonstrated that DPP4 inhibition improves survival and myocardial function after infarction by increasing regenerative capacity, particularly when potentiated by concomitant administration of G-CSF [15]. Clinically available gliptins (e.g., sitagliptin, vildagliptin) and G-CSF have been shown to effectively mobilize CD34^+^/CD45^+^ chemokine receptor (CXCR4)^+^ and CD34^+^CD45^+^ c-kit^+^ stem cells into peripheral blood. While cells release SDF-1α, however, SDF-1α is cleaved largely via DPP4 and thus cannot induce significant differentiation within the myocardial tissue. Therefore, a combined therapeutic approach aiming to increase SDF-1α release and to inhibit its proteolytic cleavage appears promising (Fig. 1). However, a previously conducted trial on combined therapy with G-CSF and sitagliptin failed to improve patients’ outcome [16, 17]. Considering the previous study, trial design, drug, and drug doses were adapted for the REC-DUT-002 trial.

### Objectives {7}

The primary objective of the study is to evaluate the efficacy of dutogliptin in combination with filgrastim in patients with STEMI compared to placebo. Efficacy is determined by change of left ventricular ejection fraction (LV-EF) from baseline to day 90 assessed by cardiac magnetic resonance imaging (cMRI). Additional, safety, tolerability, and secondary efficacy objectives will be determined. Furthermore, the trial aims to determine the pharmacokinetics (PK) and pharmacodynamics (PD) of dutogliptin in a subset of the study patients.

### Trial design {8}

**Fig. 2:**
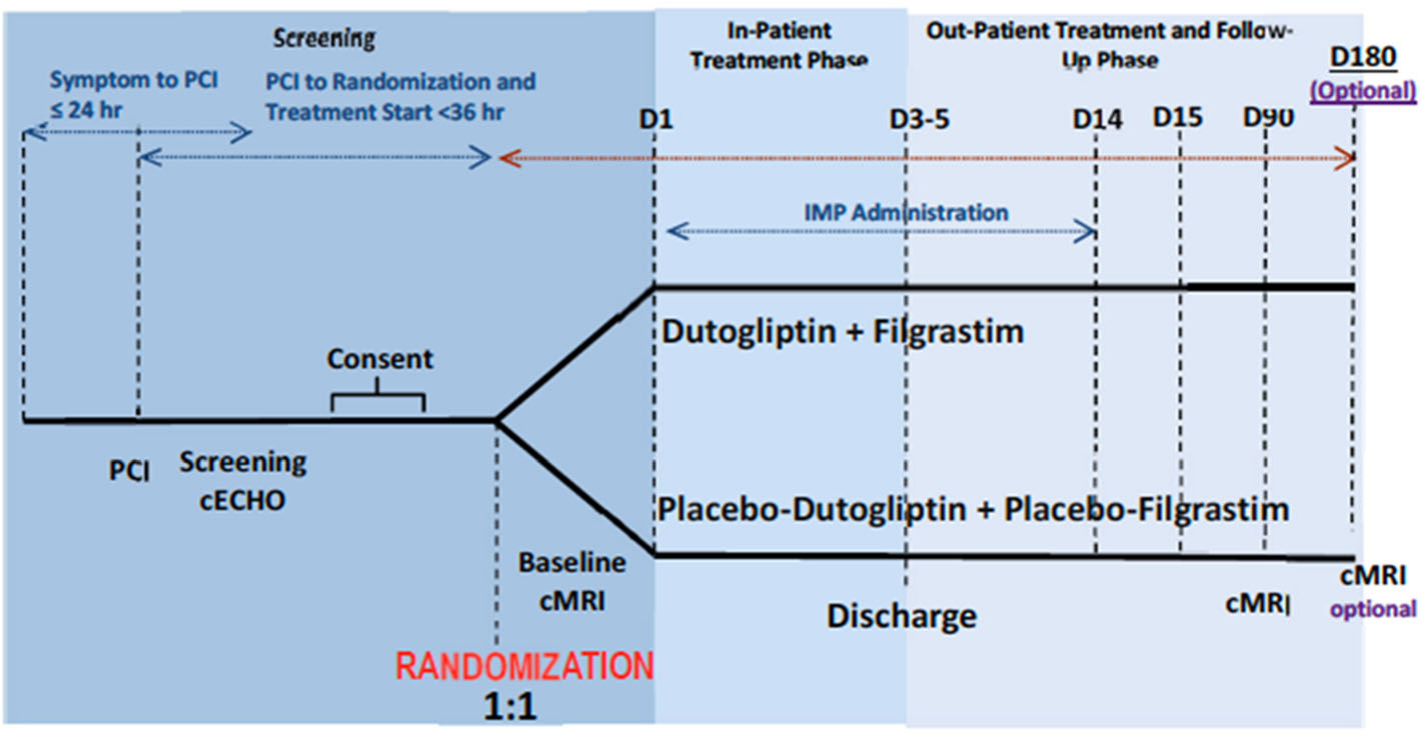
Study design

REC-DUT-002 is a multicenter, international, randomized, double-blind, placebo-controlled, phase 2 trial designed to evaluate safety and tolerability of dutogliptin (14 days) in combination with filgrastim (5 days) compared to placebo (Fig. 2).

## Methods: participants, interventions and outcomes

### Study setting {9}

The approved study protocol and related materials such as consent forms, case report forms (CRFs), and study kits have been submitted to the 12 participating centers (Table S1; university hospitals in Austria, Belgium, Hungary, Netherlands, and Poland). Nursing service is partially provided by the centers and in some centers via an external nursing service. This study is being conducted in full conformity with the 1964 Declaration of Helsinki and all subsequent revisions as well as in accordance with the guidelines laid down by the International Conference on Harmonisation for Good Clinical Practice (ICH GCP E6 guidelines).

### Eligibility criteria {10}

Patients with confirmed STEMI are assessed for eligibility. Eligible patients undergo local standard of care procedures including PCI and stent implantation (bare metal or drug-eluting). The allowable time between onset of STEMI symptoms and stent implantation (time of first balloon inflation) is up to 24 h. In order to increase the likelihood of a beneficial treatment effect with filgrastim/dutogliptin, inclusion criteria include reduced LV-EF (≤ 45%) derived from 2D echocardiography. Inclusion and exclusion criteria are provided in Table S2.

### Who will take informed consent? {26a}

Informed consent (IC) is obtained by local principal investigators or their sub-investigators. IC must be obtained prior to performing any study-specific procedures that are not standard of care and is subject to prior confirmation that inclusion/exclusion criteria during the pre-screening review are met for the enrolment of the patient.

### Additional consent provisions for collection and use of participant data and biological specimens {26b}

As part of a pharmacodynamic/pharmacokinetic substudy, a subset of patients will consent to additional blood samples being taken during the study. The consent includes the additional drawings of blood and the analysis of plasma dutogliptin concentration profiles (PK), trough dutogliptin concentrations, maximum plasma concentration (Cmax), time corresponding to Cmax, area under the drug concentration-time curve, potential additional parameters which may also be determined (volume of distribution, clearance and terminal phase half-life), plasma DPP4 activity (PD)), maximum effect (Emax), and trough plasma DPP4 activity (Table 1).

**Table 1:**
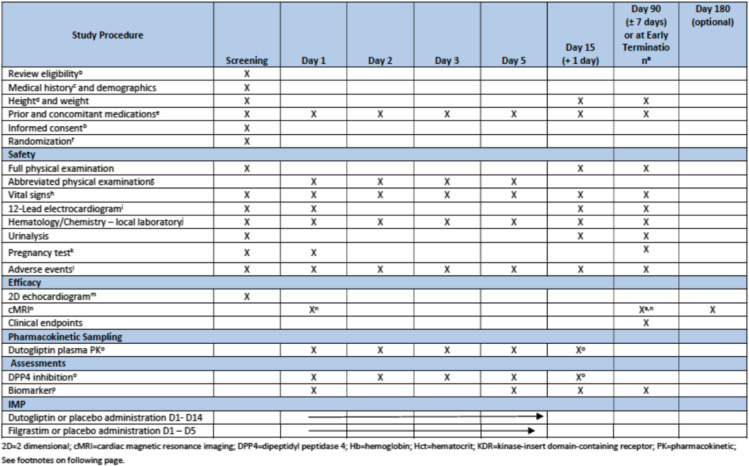
Time and events

*2D* two dimensional, *cMRI* cardiac magnetic resonance imaging, *DPP4* dipeptidyl peptidase-IV, *Hb* hemoglobin, *Hct* hematocrit, *KDR* kinase-insert domain-containing receptor, *PK* pharmacokinetic. See footnotes on the following page

## Interventions

### Explanation for the choice of comparators {6b}

NaCl was chosen as placebo considering no effects at all at the dose used.

### Intervention description {11a}

The investigational medicinal products (IMPs) dutogliptin, filgrastim, and matching placebo are being supplied as vials for subcutaneous injection—dutogliptin, 200 mg single-use vial (100 mg/mL); filgrastim (Neupogen), 480 μg single-use vial (300 μg/mL). Corresponding placebo vials are of identical design. Subjects must be randomized and initiate IMP within 36 h after stent implantation. The IMP should be administered at approximately the same time each day with a minimum of 10 h between the two daily dutogliptin doses and 16 h between filgrastim doses. Subjects are not allowed to self-administer the IMP. If a subject misses a dose of IMP, the next planned dose should be administered as scheduled. If a subject misses 2 or more doses, the investigator should be notified immediately by the hospital or homecare nursing service staff and the medical monitor should be consulted. The inpatient portion of the IMP is administered subcutaneously by the site hospital staff at the day 1 visit and while the patient remains hospitalized. Upon discharge, the homecare nursing service injects all remaining doses during home visits. The homecare nursing service is blinded to study allocation.

### Criteria for discontinuing or modifying allocated interventions {11b}

A subject may be withdrawn from the study for any of the following reasons: (1) withdrawal of consent for IMP administration; (2) withdrawal of consent from the study and all follow-up procedures; (3) non-compliance (defined as refusal or inability to adhere to the study procedures); (4) pregnancy while receiving IMP; (5) use of any other investigational treatment; (6) withdrawal due to the relevant AE at the request of the sponsor, regulatory agencies, or Institutional Review Board (IRB)/Independent Ethics Committee (IEC); (7) lost to follow-up; and (8) study termination by the sponsor. Subjects may also be withdrawn from study treatment due to elevations in liver enzymes. If a subject prematurely discontinues treatment or discontinues study participation at any time prior to completion of the day 90 visit, the subject should still return to clinic for an Early Termination visit.

### Strategies to improve adherence to interventions {11c}

Every dose of the study drug is administered by the study team. Therefore, adherence is substantially improved and accurately reported.

### Relevant concomitant care permitted or prohibited during the trial {11d}

Sexually active female subjects of childbearing potential (i.e., women who are not postmenopausal [postmenopausal is defined > 12 months amenorrhea] or who have not had a bilateral oophorectomy, hysterectomy, or tubal ligation) and all male subjects (who have not been surgically sterilized by vasectomy) must agree to use highly effective contraception during the study.

### Provisions for post-trial care {30}

An insurance for all patients with respect to covering all study-related harm was contracted by the sponsor.

### Outcomes {12}

The primary objective of the study is to evaluate the efficacy of dutogliptin in combination with filgrastim in patients with STEMI compared to placebo. Efficacy is determined by change of left ventricular ejection fraction (LV-EF) from baseline to day 90 assessed by cardiac magnetic resonance imaging (cMRI). Secondary objectives include the assessment of the pharmacokinetics (PK) and pharmacodynamics (PD; plasma dipeptidyl peptidase-IV [DPP4] activity) of dutogliptin in a subset of the study population, change from baseline in plasma biomarkers N-terminal pro-b-type natriuretic peptide (NT-proBNP) and high sensitivity troponin, and additional cardiac functional parameters derived by central, blinded review of cMRI such as (1) left ventricular end systolic volume (LVESV) (absolute and indexed), (2) left ventricular end diastolic volume (LVEDV) (absolute and indexed), (3) infarct size by cardiac magnetic resonance (cMR), (4) left ventricular mass (absolute and indexed), and (5) regional wall motion abnormalities. Safety and tolerability assessments include reporting of AEs and serious AEs (SAEs), clinical laboratory tests, ECGs, vital signs, and physical examinations.

### Participant timeline {13}

### Sample size {14}

Previous experimental data in mice showed highly significant improvement of LV-EF after myocardial infarction in G-CSF/DPP-IV inhibitor-treated animals [15]. To detect a difference of 3.8% in mean change in LV-EF from baseline to 90 days between the two treatment groups with a power of 80% using a *t* test under a significance level of 5%, 55 patients per group are needed (110 in total). For the analysis, a standard deviation of 7.0 was assumed. To compensate a dropout rate of approx. 20%, 140 patients will be randomized in total. Analyses will be based on either the intention-to-treat (ITT) population including all randomized patients or the per protocol population (PPP) including all patients in the ITT population who have completed treatment with IMP and have an adequate cMR at baseline and day 90 (month 3) without major protocol deviations. Final decisions on all protocol violations will be made on a case by case decision in a blind data review meeting before database closure. Additionally, safety will be analyzed including all subjects who have received at least 1 dose of IMP, and pharmacokinetics/pharmacodynamics will be evaluated in a subset of subjects of the ITT population.

### Recruitment {15}

A sufficient number of experienced sites have been chosen to conduct the trial. Homecare nursing service is available at all centers to maintain drug administration after hospital discharge.

## Assignment of interventions: allocation

### Sequence generation {16a}

After given written informed consent and successful screening, patients are randomized (stratified by study site) to receive either dutogliptin in combination with filgrastim or matching placebo using an interactive web response system (IRT).

### Concealment mechanism {16b}

Randomization as well as allocation of treatment and treatment kits is managed via the eCRF. Upon randomization, a unique treatment code is provided and identifies the treatment kit to use.

### Implementation {16c}

Patients will be enrolled by the investigators or study coordinators and assigned to their medication using an interactive web response system (IRT).

## Assignment of interventions: blinding

### Who will be blinded {17a}

This is a double-blind study. Subjects and study staff remain blinded to treatment assignments for the duration of their involvement in this study. The homecare nursing service will also be blinded to study allocation. Only the data management company is unblinded. As white blood count (WBC) is increased by the study medication, study staff is asked to refrain from these values during the study period.

### Procedure for unblinding if needed {17b}

The investigator will have unrestricted and immediate access to unblind the treatment code through the eCRF system.

## Data collection and management

### Plans for assessment and collection of outcomes {18a}

Safety assessment consists of clinical parameters and laboratory tests. Clinical parameters include physical examination, 12-lead electrocardiograms, and documentation of adverse events (recurrent non-fatal MI, non-fatal stroke, death due to any cause, CV death, stent thrombosis, or CHF hospitalization evaluated at day 90). Major adverse cardiac events (MACE) are defined as the combined clinical endpoints including non-fatal MI, non-fatal stroke, CV death, stent thrombosis, and CHF hospitalization. Time to cardiovascular event is defined as the time from randomization to the first occurrence of recurrent non-fatal MI, non-fatal stroke, death due to any cause, stent thrombosis, and CHF hospitalization. Safety laboratory tests for this study (chemistry, hematology, and urinalysis) are performed at local laboratories. Chemistry includes alanine transaminase, aspartate aminotransferase, bicarbonate, bilirubin, blood urea nitrogen, calcium, chloride, creatinine, glucose, potassium, sodium, and uric acid. Hematology parameters are hematocrit, hemoglobin, mean corpuscular volume, platelet count, white blood cell count (WBC), and red blood cell count (RBC). Urinalysis includes pH, specific gravity, protein (qualitative), glucose (qualitative), ketones (qualitative), bilirubin (qualitative), urobilinogen, occult blood, hemoglobin, and cells.

#### Cardiac magnetic resonance imaging

cMR is performed at 72 h and 3 months post-treatment using either a 1.5- or 3.0-T platform. Cine imaging with steady-state free precession (SSFP) and late gadolinium enhancement (LGE) imaging is performed in long-axis views and consecutive short-axis slices covering the entire left ventricle from base to apex. Typical parameters for SSFP imaging include repetition time of 3.0 ms, echo time of 1.5 ms, flip angle of 60°, temporal resolution of 35 ms, in-plane resolution of 1.4–1.7 mm, slice thickness of 7 mm, and 3-mm gap. The LGE cMR is performed using a segmented inversion recovery sequence (in-plane spatial resolution 1.7–1.4 mm; temporal resolution 160 to 200 ms) 10 min after contrast administration (gadoterate meglumine [Dotarem], Guerbet Inc; 0.1 mmol/kg). Inversion times are adjusted in the standard fashion to null viable myocardium, typically 280 to 360 ms. To evaluate myocardial edema (area at risk—AAR), a breath-hold, black blood, short-T1 triple inversion recovery pulse sequence is applied with the following parameters: repetition time (TR), 2× R-to-R intervals, echo time (TE) of 65 ms (Philips scanners, 100 ms) and inversion time (TI) of 140 ms, field of view 34 to 38 cm, and matrix 256 × 256 (or 256 × 192). Short-axis T2 images are acquired using the same slice thickness/gap as the cines and late enhancement images. Quantitative and qualitative analyses are performed offline blinded to patient details using Circle CVi42 software (v. 5.9.3, Circle Cardiovascular Imaging, Calgary, Canada) by an experienced central cMR core lab (Cardiac Imaging Research, South Australia Health Medical Research Institute, Adelaide, Australia). LV volumes and function are calculated. Infarct size is quantified semi-automatically on LGE imaging using the 5 standard deviation method (5SD technique) such that infarct size is expressed relative to ventricular volume (IS/LV), final infarct size as mass (g), and as myocardial salvage index (MSI).

### Plans to promote participant retention and complete follow-up {18b}

If a subject withdraws consent for study treatment only, the subject should be scheduled for an Early Termination visit, to occur at approximately day 15. For subjects who prematurely discontinue study treatment or study participation, SAEs that occur within 30 days after the last dose of IMP should be reported.

### Data management {19}

The investigators document subject data in local subject files. These subject files will serve as source data for the study. All eCRF data required by this protocol is recorded by investigative site personnel. All data entered into the eCRF is supported by source documentation. The documentation related to the validation of the eCRFs is maintained in a Trial Master File (TMF) which itself is maintained by the CRO and the sponsor. The eCRFs are reviewed periodically for completeness and acceptability by sponsor personnel to ensure 100% source data verification (SDV). Access to the electronic data capture system is password-protected and will be removed from the study site at the end of the site’s participation in the study. Data from the eCRF will be archived at that time as a durable record of the site’s eCRF data.

### Confidentiality {27}

Prior to study participation, the investigator shall inform the subject that the monitor(s), auditor(s), IRB/IEC, and the regulatory authorities will be granted direct access to the subject’s original medical records for verification of clinical study procedures and/or data, without violating the confidentiality of the subject, to the extent permitted by the applicable laws and regulations and that, by signing the ICF, the subject is authorizing such access. In addition, prior to study participation, the subject must be informed that the records identifying the subject will be kept confidential and, to the extent permitted by the applicable laws and/or regulations, will not be made publicly available; if the results of the study are published, the subject’s identity will remain confidential.

### Plans for collection, laboratory evaluation, and storage of biological specimens for genetic or molecular analysis in this trial/future use {33}

No biological specimens will be collected for future genetic or molecular analysis.

## Statistical methods

### Statistical methods for primary and secondary outcomes {20a}

The statistical analysis will be conducted by Assign Data Management and Biostatistics GmbH. Statistical analysis is based on the International Conference on Harmonisation (ICH) Guidelines “Structure and Content of Clinical Study Reports” and “Statistical Principles for Clinical Trials.” For all analyses, SAS® (version 9.3 or higher; SAS Institute Inc., Cary, NC, USA) will be used. Data will be summarized by treatment group and, where appropriate, by visit. Descriptive statistics (number of observations, mean, standard deviation, minimum, median, maximum) will be provided for continuous variables. Frequency counts and percentages will be presented for categorical variables. Incidence rates for treatment-emergent AEs (TEAEs) will be summarized overall, by maximum severity, and by relationship to IMP for each treatment group. Rates will be compared between the two groups by means of Fisher’s exact test.

Changes from baseline in LV-EF, LVESV, LVEDV, infarct size, left ventricular mass, and regional wall motion will be evaluated for statistical significance using an analysis of covariance model with randomization stratification factors as covariates. All data exclusions, including premature terminations, will be detailed and tabulated. Data listings will include all enrolled subjects. A two-sided significance level of 5% will be applied; two-sided 95% confidence intervals will be calculated. A detailed statistical analysis plan including pre-specified subgroup analyses is still under review and will be finalized ahead of database lock.

### Interim analyses {21b}

A safety analysis is scheduled after randomization and active treatment of 30 patients. A database snapshot will be performed when all subjects have completed day 90, and the data will be analyzed. This is the primary analysis. Investigators and subjects remain blinded. A final database closure will be performed when the subjects agreeing to the day 180 assessment have completed the tests. A secondary analysis of the resulting additional data will then be performed. All these analyses will be performed by the statisticians of the data and safety monitoring board (DSMB).

### Methods for additional analyses (e.g., subgroup analyses) {20b}

Subgroup analyses are planned but not yet decided.

### Methods in analysis to handle protocol non-adherence and any statistical methods to handle missing data {20c}

Missing values will generally not be imputed.

### Plans to give access to the full protocol, participant level-data, and statistical code {31c}

The datasets analyzed during the current study are available from the corresponding author on reasonable request.

## Oversight and monitoring

### Composition of the coordinating center and trial steering committee {5d}

The steering committee consists of the authors Dirk von Lewinski, Joseph B Selvanayagam, Richard A Schatz, Bernd Jilma, Jacek Kubica, Tom Povsic, Darrell Nix, and Stephan Henauer. DN and SH are employees of the sponsor; all other members are independent of the sponsor. The committee members helped to design the study and support the ongoing trial with advice or as active investigators or both.

There is no trial steering committee (TSC) but only a scientific advisory board (SAB) in this sponsored trial.

### Composition of the data monitoring committee, its role, and reporting structure {21a}

The data safety monitoring board is independent of the sponsor. It includes statisticians and is chaired by Prof. Andrew Coates.

Local organization including identifying potential recruits and taking consent is at the responsibility of the local PIs.

### Adverse event reporting and harms {22}

The investigator must make every effort to properly evaluate all information relevant to the reported AE in such a way that a diagnosis can be confidently made and reported. When recording and/or reporting AEs or SAEs, the following elements must be included: (1) the fulfilled criteria for seriousness, (2) the severity of the event, and (3) the relationship of the event to study treatment. The data management safety board supervises these responsibilities.

### Frequency and plans for auditing trial conduct {23}

An audit may be performed independently of, and separately from, routine monitoring to evaluate clinical study conduct and compliance with the protocol, SOPs, GCP, and the applicable regulatory requirements.

### Plans for communicating important protocol amendments to relevant parties (e.g., trial participants, ethical committees) {25}

Protocol amendments will be discussed in the Scientific Board, and the sponsor will be notified first. Then, the PI will notify all centers, and a copy of the revised protocol will be sent to the local PIs to add to the Investigator Site File. Deviations from the protocol will be fully documented using a report form.

### Dissemination plans {31a}

Trial data and results will be made available via EudraCT. In addition, publication in scientific journals is planned and will include the interim safety analysis data, the PK/PD data, and the final results of the study. Outcomes will also be presented at national and international scientific meetings. Whether the study is completed or prematurely terminated, the clinical study report will be prepared and provided to the regulatory agencies as required by the applicable regulatory requirement(s).

## Discussion

Augmenting evidence indicates short-term [12] beneficial effects of G-CSF therapy after large myocardial infarction. Controversial data and moderate effects may likely be explained by inadequate concentrations of SDF-1α at the site of infarction. This deficit might be overcome by simultaneous DPP4 inhibition. Therefore, the randomized, double-blinded REC-DUT-002 trial aims to investigate safety and tolerability of the new small molecule DPP-IV inhibitor dutogliptin in combination with G-CSF (filgrastim) in patients with STEMI.

Preclinical studies have demonstrated that G-CSF-based stem cell mobilization in combination with genetic or pharmaceutical CD26/DPP4 inhibition after STEMI results in improved cardiac homing of stem cells, enhanced heart function, and increased survival. In mice, combining genetic and pharmacologic inhibition of DPP4 with G-CSF-mediated stem cell mobilization after MI led to (1) decreased myocardial DPP4 activity, (2) increased myocardial homing of circulating CXCR-4^+^ stem cells, (3) reduced cardiac remodeling, and (4) improved heart function and survival [15, 18].

The combination of DPP4 inhibitor sitagliptin and G-CSF was previously tested in the SITAGRAMI trial [16]. This trial revealed overall safety of the combined therapy but was neutral with regard to efficacy. However, in contrast to the REC-DUT-002 trial, treatment started considerably later (up to 6 days after PCI compared to < 36 h), baseline LV-EF was almost normal (average of 52% in SITAGRAMI compared to < 45% in RECARDIO), NSTEMI patients were also included, and the DPP4 inhibitor sitagliptin was administrated orally and at a questionable dose instead of the subcutaneous administration employed in REC-DUT-002. Early timing of the therapy appears to be especially crucial and is therefore of high importance in the REC-DUT-002 study protocol.

The safety profile of dutogliptin can be expected to be good. So far, 12 clinical studies with oral dutogliptin have been conducted. The largest placebo-controlled phase 2b study of dutogliptin (200 mg or 400 mg daily) in 423 patients with diabetes mellitus [19] revealed adverse event rates comparable to placebo. Only a slightly increased frequency of adverse events (AEs) (headache, arthralgia, sinusitis, and dizziness (≤ 2.5% over placebo)) was recorded in the 400-mg dutogliptin group. Conversely, minor increases in frequency of upper respiratory tract infection, influenza, and hematuria were noted in the placebo group and no SAEs were reported in either group. An analysis of vital signs (blood pressure, respiratory rate, body temperature) did not show any clinically relevant differences between the treatment groups and placebo. Similarly, there were no relevant changes in hematology and chemistry parameters, including lipids.

In a phase 1 study [20] in healthy volunteers, dutogliptin was administered subcutaneously at doses ranging from 30 to 120 mg/day. Inhibition of plasma DPP4 increased in duration with increasing dose. However, complete (≥ 80%) inhibition could not be achieved for 24 h. Sustained strong inhibition was observed to last for 8–12 h following a single 60 mg dose, and consequently, twice daily 60 mg dosing was selected as the active dose for this study.

STEMI is a major cause for developing heart failure and associated with poor quality of life and increased mortality. Regenerative therapies might reduce this burden in the future. The REC-DUT-002 trial is the first trial to test dutogliptin in combination with G-CSF in patients with STEMI. Besides examining safety and tolerability of the combined therapy, results will lay the foundation for an appropriately powered cardiovascular outcome trial to test the efficacy of this combined pharmacological strategy in patients with STEMI.

A potential limitation of treatment allocation blinding is the pronounced effect of filgrastim on the white blood count (WBC). Despite the fact that a certain increase in leucocytes is common in MI patients due to inflammatory processes, the increase due to filgrastim is considerably stronger. Detection of pronounced increases in WBC is therefore suggestive for patients in the verum group. Study group members are therefore asked to refrain from checking on the WBC during the study period.

## Trial status

At present, 12 sites are actively recruiting patients, and it is anticipated the trial will be completed in 2020. The first patient was consented and randomized on December 6, 2018; as of November 30, 2019, 25 patients have been randomized. The protocol is version 1.3 (October 10, 2019). Estimated date of recruitment completion is December 2020.

## Supplementary information

**Additional file 1: Table S1.** List of study centers and investigators of the REC-DUT-002 trial. **Table S2.** List of inclusion and exclusion criteria.

## Abbreviations

cMRI: Cardiac magnetic resonance imaging
CRFs: Case report forms
DPP4: Dipeptidyl peptidase-IV
G-CSF: Granulocyte-colony stimulating factor
ITT: Intention-to-treat
LV-EF: Left ventricular ejection fraction
LVEDV: Left ventricular end diastolic volume
LVESV: Left ventricular end systolic volume
MACE: Major adverse cardiac events
NSTEMI: Non-ST-elevation myocardial infarction
NT-proBNP: N-terminal pro-b-type natriuretic peptide
PCI: Percutaneous coronary intervention
PD: Pharmacodynamics
PK: Pharmacokinetics
PPP: Per protocol population
SDF-1α: Stromal cell-derived factor 1 alpha
STEMI: ST-elevation myocardial infarction
