# Supplementary material for: “Protocol for a phase 2, randomized, double-blind, placebo-controlled, safety and efficacy study of dutogliptin in combination with filgrastim in early recovery post-myocardial infarction”: study protocol for a randomized controlled trial
Source: Trials. 2020 Aug 26;21:744. doi: 10.1186/s13063-020-04652-0 (PMC7448478; doi:10.1186/s13063-020-04652-0)
Supplement: Supplementary file 1 — Additional file 1: Table S1. List of study centers and investigators of the REC-DUT-002 trial. Table S2. List of inclusion and exclusion criteria. [file 13063_2020_4652_MOESM1_ESM.docx]

**Table S1: List of study centers and investigators of the RecDut-002 trial.**

**Austria**

- Clinical Department of Cardiology, Graz, Austria
- Klinikum Klagenfurt am Wörthersee, Klagenfurt, Austria

**Belgium**

- Algemeen Stedelijk Ziekenhuis Aalst, Aalst, Belgium

**Hungary**

- Military Hospital, Budapest, Hungary
- Semmelweis Egyetem Városmajori Szív- és Érgyógyászati Klinika, Budapest, Hungary
- Debreceni Egyetem Kardiológiai és Szívsebészeti Klinika, Debrecen, Hungary
- Borsod-Abaúj-Zemplén Megyei Központi Kórház és Egyetemi Oktatókórház, Miskolc, Hungary

**Netherlands**

- Maasstad Ziekenhuis, Rotterdam, Netherlands

**Poland**

- Nicolaus Copernicus University, Bydgoszcz, Poland
- SPS Szpital Zachodni, Grodzisk Mazowiecki, Poland
- Samodzielny Publiczny Szpital Wojewódzki im. Papieża Jana Pawła II, Zamość, Poland
- Centralny Szpital Kliniczny Uniwersytetu Medycznego w Lodzi ul. Pomorska 251, Łódź, Poland

**Table S2: List of inclusion and exclusion criteria**

Inclusion/Exclusion Criteria

To be eligible for this study, subjects must meet ALL of the following inclusion criteria:

- Male or female, age 18 to 85 (having reached 18 and before having reached 86 at the time of ICF signing)
- Body weight <96 kg (212 lb)
- Able to provide written informed consent, including signing and dating the ICF
- Diagnosis of STEMI (defined as new ST-segment elevation at the J point of at least two continuous leads of >2 mm [0.2 mV] in men or >1.5 mm [0.1 mV] in women in leads V2 and V3 OR >1 mm in any other contiguous precordial leads or the limb leads [for both men and women]) with PCI (bare metal or drug-eluting stent) and thrombolysis in myocardial infarction flow grade 2 or 3 occurring up to 24 hours after symptom onset (to time of first balloon inflation).
- Left ventricular ejection fraction (LVEF) ≤45% obtained by cECHO performed within 36 hours post-stent placement
- Receiving standard medical therapy for post-myocardial infarction (MI) treatment, according to local procedures and the Principal Investigator’s discretion
- Female subjects of childbearing potential must have a negative serum pregnancy test at Screening and an additional negative urine pregnancy test prior to the first dose of IMP unless regulated differently by national legislation
- Sexually active female subjects of childbearing potential (i.e. women who are not postmenopausal or who have not had a bilateral oophorectomy, hysterectomy, or tubal ligation) and all male subjects (who have not been surgically sterilized by vasectomy) must agree to use highly effective contraception during treatment and for 4 weeks after the last dose.

Subjects who meet ANY of the following exclusion criteria must be excluded from the study:

- Previous MI prior to Screening
- Complex peri/post-MI clinical course, including arrhythmias, cardiogenic shock, pulmonary edema requiring mechanical ventilation, or requirement for vasopressor medications
- Significant pre-existing cardiomyopathy with known LVEF ≤45% or moderate to severe mitral or aortic valvular disease
- Amyloidosis, hypertrophic obstructive cardiomyopathy, restrictive cardiomyopathy, or constrictive pericarditis
- Existing heart transplant
- Ventricular tachycardia or fibrillation not associated with an acute ischemic episode
- Uncontrolled hypertension (systolic >180 mmHg or diastolic >120 mmHg)
- Treatment with any DPP4 inhibitors (e.g., alogliptin, linagliptin, vildagliptin, saxagliptin, sitagliptin) or granulocyte-colony stimulating factor (G-CSF) medication (e.g., filgrastim, lenograstim, pegfilgrastim, lipegfilgrastim) within 4 months prior to Randomization
- Contraindication to treatment with filgrastim, including known allergy to filgrastim or other G-CSF medication
- Anemia defined as hemoglobin <9 g/dL prior to randomization
- Thrombocytosis (platelets >500 k/μL)
- Known positive serology for hepatitis B, hepatitis C, or human immunodeficiency virus, any other indication of liver disease or injury
- Alanine aminotransferase (ALT) concentrations >3 times the upper limit of normal (ULN) and (total bilirubin >2 x ULN, or INR >1.5) prior to Randomization, according to local laboratory assessments, and/or any indication of liver disease or injury. If ALT>3 and all other criteria are met, the test may be repeated within the time window before randomization.
- History of cirrhosis and Child-Pugh score B or C
- Current fever greater than 101.4°F (38.6°C) or recent systemic infection within 2 weeks prior to randomization
- Contraindication to cMRI procedure, including prior implantable cardioverter defibrillator placement, known reaction to gadolinium, claustrophobia, non-MRI-compatible, cochlear implant, morbid obesity, or presence of ferromagnetic material including shunts, shrapnel, penile prostheses, or blood vessel coil
- Pregnant, planning to become pregnant, or nursing female subjects
- Autoimmune disease requiring immunosuppressive therapy or chronic steroid treatment >5 mg/day prednisolone or equivalent
- Significant renal impairment defined as estimated glomerular filtration rate <45 mL/min/1.73 m^2^, using the Chronic Kidney Disease Epidemiology Collaboration equation
- Active neoplasm requiring surgery, chemotherapy, or radiation within the past 12 months (subjects with a history of malignancy who have undergone curative resection or otherwise not requiring treatment for at least 12 months prior to Screening with any detectable recurrence are allowed)
- Malignant hematological disease, i.e., chronic myeloid leukemia or myelodysplastic syndrome
- History of cerebrovascular accident or transient ischemic attack in the past 6 months
- History of pneumonia in the last 4 weeks
- History of any significant medical or psychiatric disorder that in the opinion of the Investigator would make the subject unsuitable for participation in the study
- Treatment with an investigational drug within 30 days or five half-lives (whichever is longer) or treatment with an investigational biologic drug within 6 weeks prior to Randomization
- Participation in another concurrent clinical trial involving a therapeutic intervention (participation in observational studies and/or registry studies is permitted)
- Unable or unwilling to comply with the requirements of the study
- Subject and/or an immediate family member is an employee of the investigational site directly affiliated with this study, the sponsor or the contract research organization
- Considered by the Investigator to be unsuitable to participate in the study for any other reason
- Persons who are in an institution as a result of an administrative or judicial order, or soldiers
- History of alcohol or drug abuse
